# Physical Examination Identification in Medical Education Videos: Zero-Shot Multimodal AI With Temporal Sequence Optimization Study

**DOI:** 10.2196/76586

**Published:** 2025-12-18

**Authors:** Shinyoung Kang, Michael Holcomb, David Hein, Ameer Hamza Shakur, Thomas Dalton, Andrew Jamieson

**Affiliations:** 1 Lyda Hill Department of Bioinformatics The University of Texas Southwestern Medical Center Dallas, TX United States; 2 Department of Internal Medicine The University of Texas Southwestern Medical Center Dallas, TX United States

**Keywords:** artificial intelligence, AI, medical education, multimodal large language models, video segmentation

## Abstract

**Background:**

Objective structured clinical examinations (OSCEs) are widely used for assessing medical student competency, but their evaluation is resource-intensive, requiring trained evaluators to review 15-minute videos. The physical examination (PE) component typically constitutes only a small portion of these recordings; yet, current automated approaches struggle with processing long medical videos due to computational constraints and difficulties maintaining temporal context.

**Objective:**

This study aims to determine whether multimodal large language models (MM-LLMs) can effectively segment PE periods within OSCE videos without previous training, potentially reducing the evaluation burden on both human graders and automated assessment systems.

**Methods:**

We analyzed 500 videos from 5 OSCE stations at University of Texas Southwestern Simulation Center, each 15 minutes long, by using hand-labeled PE periods as ground truth. Frames were sampled at 1, 2, or 3 seconds. A pose detection preprocessing step filtered frames without people. Six MM-LLMs performed frame-level classification into encounter states by using a standardized prompt. To enforce temporal consistency, we used a hidden Markov model with Viterbi decoding, merging states into 3 primary activities (consulting/notes, physical examination, and no doctor) and adding a brief edge buffer to avoid truncating true PE segments. Performance was computed per video and averaged across the dataset by using recall, precision, intersection over union (IOU), and predicted PE length with 95% CIs.

**Results:**

At 1-second sampling, GPT-4o achieved recall of 0.998 (95% CI 0.994-1.000), IOU of 0.784 (95% CI 0.765-0.803), and precision of 0.792 (95% CI 0.774-0.811), identifying a mean of 175 (SD 83) seconds of content per video as PE versus a mean labeled PE of 126 (SD 61) seconds, yielding an 81% reduction in video needing review (from 900 to 175 seconds). Across stations, recall remained high, with expected IOU variability linked to examination format and camera geometry. Increasing the sampling interval modestly decreased recall while slightly improving IOU and precision. Comparative baselines (eg, Gemini 2.0 Flash, Gemma 3, and Qwen2.5-VL variants) demonstrated trade-offs between recall and overselection; GPT-4o offered the best balance among high-recall models. Error analysis highlighted false negatives during occluded or verbally guided maneuvers and false positives during preparatory actions, suggesting opportunities for camera placement optimization and multimodal fusion (eg, audio cues).

**Conclusions:**

Integrating zero-shot MM-LLMs with minimal-supervision temporal modeling effectively segments PE periods in OSCE videos without requiring extensive training data. This approach significantly reduces review time while maintaining clinical assessment integrity, demonstrating that artificial intelligence methods combining zero-shot capabilities and light supervision can be optimized for medical education’s specific requirements. This technique establishes a foundation for more efficient and scalable clinical skill assessment across diverse medical education settings.

## Introduction

### Background

Objective structured clinical examinations (OSCEs) have been integral to medical education assessment since their introduction in the 1970s, offering a standardized method to assess clinical competencies [1-3]. During these examinations, medical students rotate through multiple 15-minute stations, interacting with standardized patients to demonstrate skills in communication, physical examination (PE), and clinical reasoning. This controlled setting ensures consistent evaluation across diverse scenarios such as pediatric assessments or surgical consultations. Recent bibliometric mapping shows OSCE scholarship expanding with a sustained emphasis on performance, competence, and skills, underscoring the assessment focus of grading OSCE examinations [4].

Grading OSCE performance relies heavily on trained standardized patient evaluators who review video recordings, assessing students against rubrics that emphasize verbal communication, emotional intelligence, and PE proficiency. This manual process is labor intensive and resource demanding, creating a significant bottleneck as medical schools scale training for larger cohorts.

While automated assessment has progressed for written components of medical examinations, leveraging natural language processing for transcript analysis and written exams [5-7], video-based evaluations such as OSCEs remain challenging to automate [8,9]. The complexity stems from the dynamic, visual nature of clinical interactions, including subtle movements and gestures that require deep domain understanding.

Existing multimodal models struggle with 15-minute OSCE recordings due to computational constraints and challenges maintaining temporal context over extended durations as models must either truncate sequences or aggressively downsample, sacrificing critical details [10-12]. These limitations are particularly pronounced for PEs, where students’ practical skills must be precisely evaluated. Therefore, PE assessment remains a critical gap in automated OSCE evaluation, and identifying and isolating these segments is a foundational step toward comprehensive automated assessment of clinical competencies. Moreover, emerging evidence that large language models can handle complex multimodal clinical cases further motivates our choice of multimodal large language models (MM-LLMs) for video understanding [13].

### Significance

To address this gap, we propose a novel approach that fundamentally differs from general video classification methods. Unlike traditional approaches that assign a single label to entire recordings [14,15], our method performs temporal segmentation to isolate specific activities within OSCE videos precisely. This distinction is critical in educational contexts, where evaluators need to assess discrete skills demonstrated in brief segments, such as the PE portion, which typically constitutes only 15% to 20% of the total recording time.

Despite the critical importance of PE assessment in medical education, to our knowledge, this study represents the first attempt to automatically segment PE periods within OSCE videos. While temporal segmentation approaches have been used in other domains [16], the unique requirements of OSCE evaluation—particularly the need for near-perfect recall to capture even brief examination components—present distinct challenges that general methods were not designed to address.

Our approach integrates MM-LLMs with Viterbi decoding through hidden Markov models (HMMs), creating a solution specifically tailored to medical education’s requirements. This combination leverages the contextual understanding capabilities of MM-LLMs for frame-level classification while applying Viterbi decoding to filter out spurious false positives and negatives, ensuring consistent segmentation of examination activities across the video timeline. Our primary research question was as follows: can MM-LLMs accurately identify PE activities in OSCE videos without specialized training across diverse examination scenarios?

We evaluated our approach across 500 recordings from 5 OSCE stations at University of Texas (UT) Southwestern Simulation Center: family medicine, surgery 1, surgery 2, internal medicine 1, and internal medicine 2. Each station presents unique challenges, from differing examination techniques to varied spatial configurations. Additionally, each station uses different camera angles due to variations in room setup, allowing us to test the robustness of our approach across diverse recording environments.

By streamlining video analysis, our method not only enhances grading efficiency but also establishes a foundation for fully automated OSCE assessment. This advancement aligns with the broader shift toward competency-based medical education frameworks, where efficient, objective assessment of clinical skills is increasingly vital. As medical education evolves to meet growing health care workforce demands, such technological innovations could significantly improve the scalability and consistency of clinical skill evaluation while alleviating institutional resource constraints.

## Methods

### Study Design and Dataset

This study analyzed OSCE videos from medical students at the UT Southwestern Simulation Center. We specifically focused on the comprehensive OSCE administered during clerkship. Of the 10 total OSCE stations administered in the fall of 2019, we selected the 5 that included PE components: family medicine, surgery 1, surgery 2, internal medicine 1, and internal medicine 2. For each station, we randomly selected 100 videos (500 videos in total), each approximately 15 minutes (900 seconds) in length.

The videos were recorded at 1920 × 1080 resolution with 2 fixed cameras: one facing the patient and one facing the medical student. For our analysis, we primarily used the camera angle facing the patient to best capture the medical student’s movements during PE.

### Ground Truth Labeling

To establish ground truth for model evaluation, a single rater with emergency medical technician–level expertise manually annotated the PE periods in all 500 videos. We defined the PE period using clear visual criteria: beginning when the medical student made direct physical contact with the patient for assessment purposes (such as when the otoscope light aligned with the patient’s eye during an eye examination) and ending when all direct physical assessment activities concluded. This definition excluded preparatory activities such as retrieving examination tools, ensuring that only unambiguous examination activities were labeled. Given the straightforward nature of identifying direct physical contact beginning and end points, a single expert annotator was sufficient for this task. To establish ground truth for model evaluation, a single rater with emergency medical technician–level expertise manually annotated the PE periods in all 500 videos, as shown in Table 1.

**Table 1:** Video count and physical examination duration statistics by station.

	Videos, n	Physical examination time (s), mean (SD)
Overall	500	126 (61)
Family medicine	100	141 (70)
Surgery 2	100	163 (74)
Internal medicine 1	100	108 (54)
Surgery 1	100	119 (59)
Internal medicine 2	100	101 (47)

### Automated PE Segmentation Workflow

**Figure 1:**
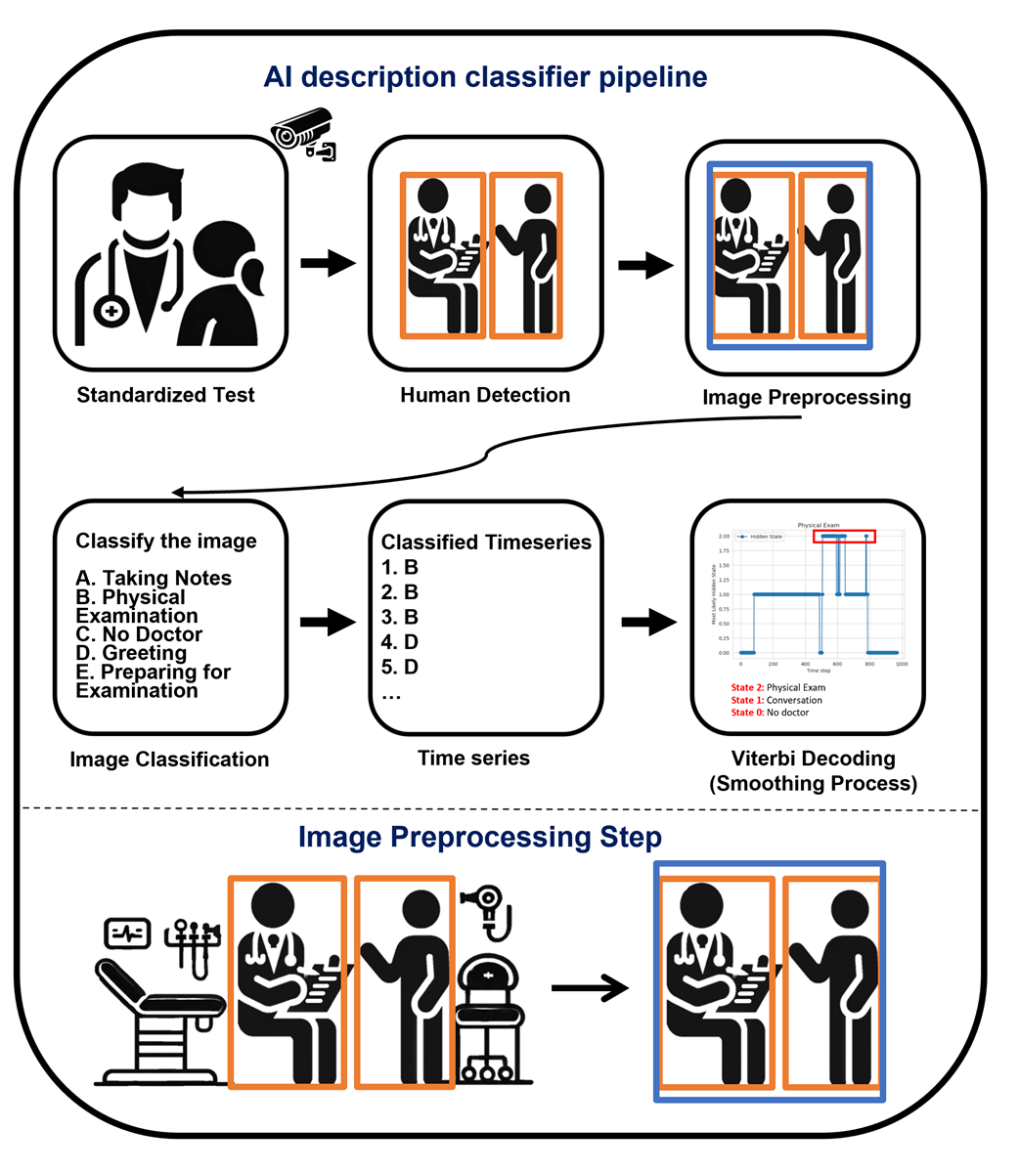
Frame classification pipeline for physical examination detection. This streamlined approach classifies frames directly into states (taking notes, physical examination, no doctor, greeting, or preparing for examination) before applying Viterbi decoding. AI: artificial intelligence.

Our approach used a streamlined pipeline for identifying PE periods within OSCE videos. This process consisted of human detection preprocessing, direct frame classification using MM-LLMs, and temporal consistency enforcement through Viterbi decoding. The complete workflow is illustrated in Figure 1.

### Preprocessing Using Human Pose Detection

We used YOLO11 (Ultralytics Inc) to detect human presence in each frame [17,18]. This preprocessing step allowed us to optimize computational efficiency by only having the MM-LLM process frames containing people. Frames without detected humans were automatically assigned to the “no doctor” state.

The model identified the bounding boxes of human skeletons and was configured to detect a maximum of 2 individuals per frame (the medical student and standardized patient), which aligned with the design of OSCE systems. We set a minimum confidence threshold of 0.5 for human detection, balancing detection sensitivity with computational efficiency.

For each frame containing humans, we created a square bounding box encompassing all detected individuals. As illustrated in Figure 1, these cropped frames were then resized to 512 × 512 pixels for MM-LLMs.

### Frame Classification Approach

We conducted frame analysis at 3 different sampling rates to evaluate computational efficiency versus accuracy trade-offs: 1 frame every second (1-second interval), 1 frame every 2 seconds (2-second interval), and 1 frame every 3 seconds (3-second interval). These specific sampling rates were selected to balance computational requirements with detection accuracy. The lower bound of 3-second intervals was particularly significant as PEs in OSCEs can sometimes be brief (2-3 seconds for specific techniques). Sampling less frequently would risk missing these short examination components entirely.

For frame classification, we evaluated 6 MM-LLMs: GPT-4o (November 6, 2024), GPT-4o-mini (July 18, 2024), Gemini 2.0 Flash (February 5, 2024), Gemma 3, Qwen2.5-VL-72B, and Qwen2.5-VL-7B. Open-source models were hosted on graphics processing unit nodes within BioHPC, UT Southwestern’s shared high-performance computing infrastructure. Proprietary models were accessed via secure, HIPAA (Health Insurance Portability and Accountability Act)- and Family Educational Rights and Privacy Act (FERPA)–compliant application programming interfaces. These models analyzed each preprocessed frame using the structured prompt shown in Textbox 1.

**Prompts for frame classification task.**

**System message—prompt**

- “You are an AI assistant designed to analyze images about medical scenarios and select the most appropriate interpretation from a set of multiple-choice options. Your responses must be in JSON format.”

**User message—prompt**

“The patient is wearing a gown.”

Choose from the following choices:

A. doctor is reviewing information, taking notes, or consulting with the patient

B. doctor is performing a physical examination

C. there is no doctor

D. the doctor is greeting the patient

E. doctor is preparing for examination

Answer in JSON format ONLY:

{‘Reasoning’: ‘a brief explanation of why this choice was selected based on what is observed in the image’

‘Choice’: ‘letter,’}

Example: {‘Reasoning’: ‘The doctor is using a stethoscope to listen to the patient’s chest, actively performing a physical examination.’

‘Choice’: ‘B,’}”

### Viterbi Decoding for State Prediction

**Figure 2:**
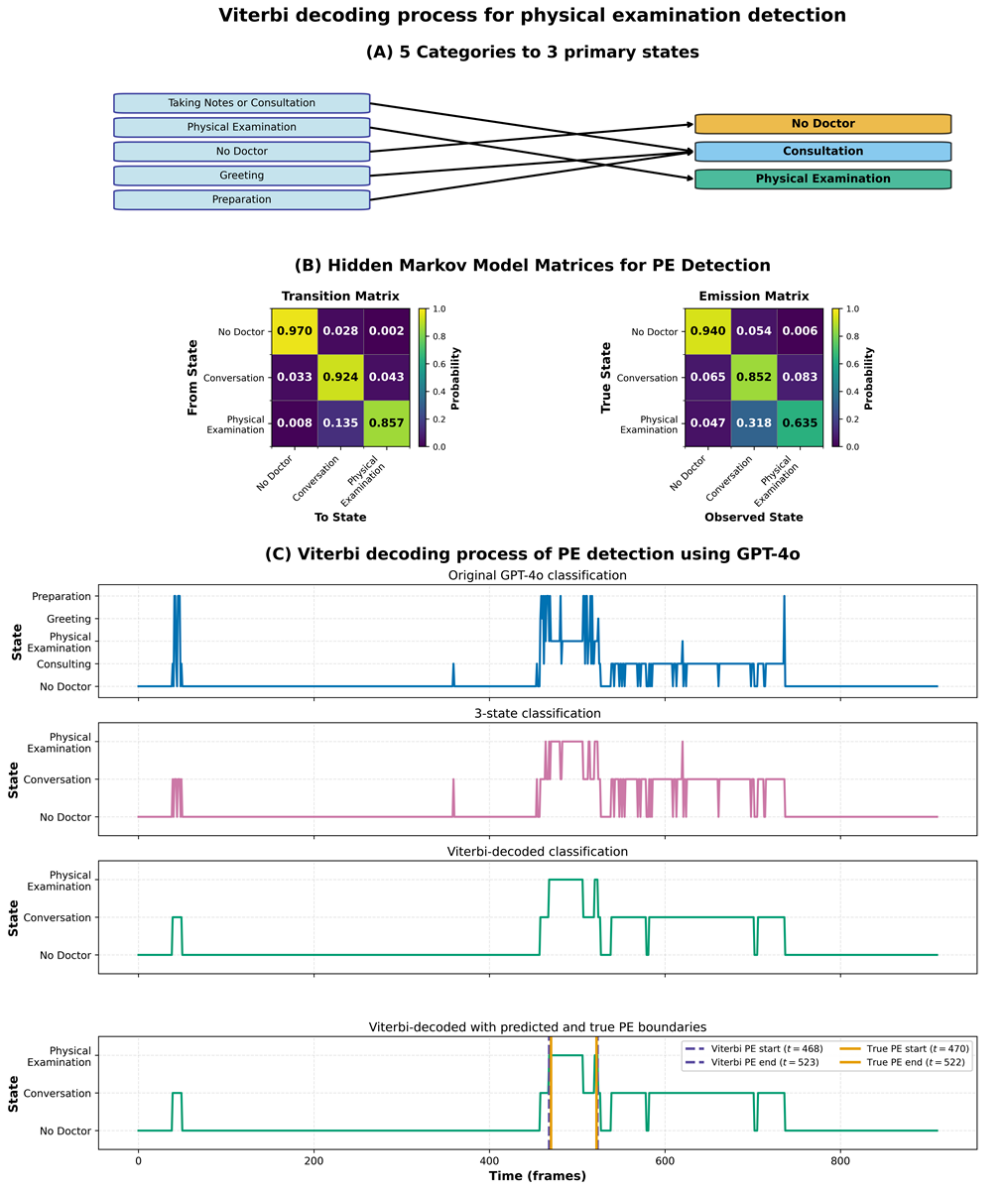
Viterbi decoding process: (A) 5 categories to 3 primary states, (B) hidden Markov model matrices—transition matrix showing probabilities between states and emission matrix showing a relationship between true and observed states, and (C) example of the Viterbi decoding process for finding a physical examination within a video. PE: physical examination.

After obtaining frame-level classifications, we implemented an HMM with Viterbi decoding to enforce temporal consistency and address the inherent noise in frame-level predictions. We simplified the analysis by merging the 5 initial categories into 3 primary states that reflect the fundamental activities in OSCE encounters (Figure 2A): taking notes/consulting, physical examination, and no doctor.

We randomly selected 10 videos from each station (50 videos in total) to calculate the average transition and emission matrices for the HMM, shown in Figure 2B. In 500 bootstrap resamples (per video), elementwise mean absolute change was 0.008 for both matrices, and 95% CIs for self-transitions averaged approximately 0.046 (Multimedia Appendix 1). The transition matrix captured the probability of moving between states, whereas the emission matrix represented the probability of observing a particular state given the true underlying state.

On the basis of the OSCE protocol, we initialized the model with “no doctor” as the starting state as sessions begin with the patient alone in the examination room. We then applied the Viterbi algorithm through the PyHHMM library [19] to determine the most likely sequence of states and identify the PE period.

**Figure 3:**
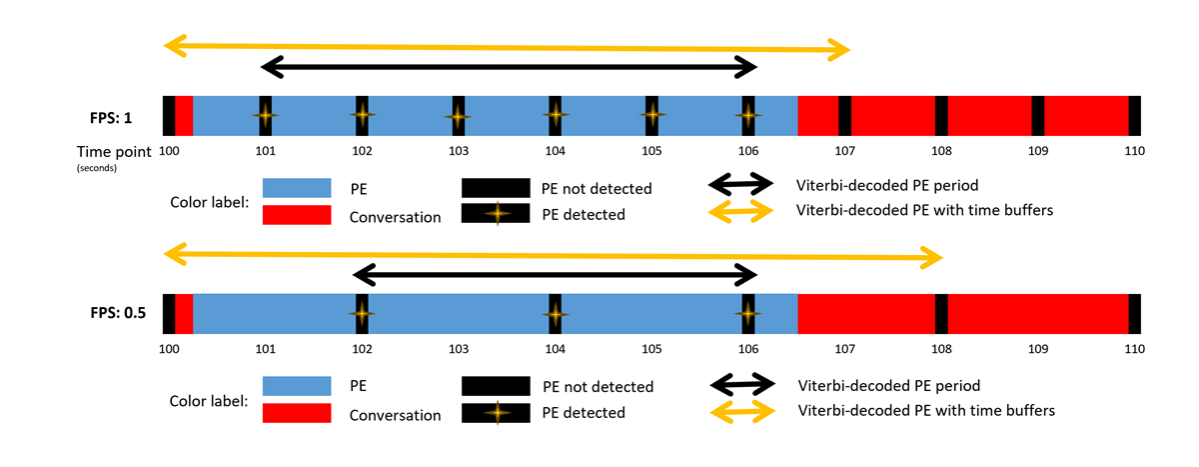
Buffer time implementation to ensure complete transition capture between sampled frames. FPS: frames per second; PE: physical examination.

To ensure complete capture of PE periods despite discrete sampling, we added a buffer time at the edges of detected periods proportional to the frame sampling rate (Figure 3). This buffer compensated for transitions that might occur between sampled frames. Additional experiments examining the effect of different buffer times (0, 15, and 30 seconds) on performance metrics are shown in Multimedia Appendix 2, demonstrating how increased buffer sizes improve recall at the expense of precision and intersection over union (IOU).

### Evaluation Metrics

We assessed model performance using 4 primary metrics calculated per video and averaged across all videos.

The first metric was recall, which is the proportion of the labeled PE period correctly captured by the model. A high recall is critical as missing any PE segment compromises clinical skill assessment:



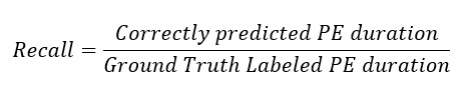



A recall of 1 constituted complete capture of the PE, whereas a recall of 0 indicated that no PEs captured.

The second metric was precision, which is the accuracy of the predicted PE segments, calculated as the ratio of correctly predicted PE duration to total predicted PE duration:



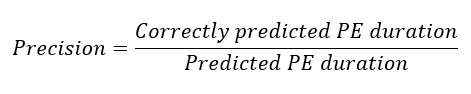



The third metric was IOU, which measures the overlap accuracy between predicted and actual PE periods:



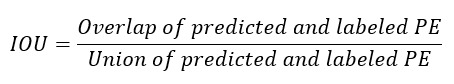



The fourth metric was predicted PE length, which evaluates practical utility by quantifying the potential reduction in video review time:



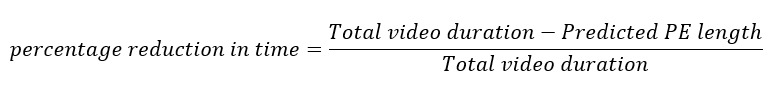



We prioritized high-recall models, accepting slightly longer predictions to ensure that critical examination segments were not omitted. All metrics were calculated on a per-video basis and averaged across the dataset, with 95% CIs reported.

### Ethical Considerations

This study was reviewed and approved by the UT Southwestern institutional review board (STU-2022-0908). The institutional review board granted a waiver of informed consent for the retrospective analysis of existing OSCE recordings, which are FERPA-protected educational records. Raw videos stayed on secure institutional servers, with access restricted to study staff; analyses were conducted on HIPAA- and FERPA-compliant systems, and only deidentified time stamps and summary metrics were used for reporting. Participants were not compensated. The manuscript and supplements do not include identifiable images or video frames, so masking was not required.

## Results

### Overall Model Performance Across Sampling Rates

Our evaluation compared hand-labeled PE periods against those identified through our approach across 5 OSCE stations (n=500 videos). We prioritized recall as our primary metric as missing examination components could lead to incomplete evaluation of clinical skills in high-stakes educational assessments. Table 2 shows the comprehensive findings across all models and sampling rates.

**Table 2:** Overall performance comparison by sampling rate.

Model and sampling rate (s)			Recall (95% CI)		IOU^a^ (95% CI)		Precision (95% CI)		PE^b^ length (s; 95% CI)		Predicted length (s; 95% CI)
**GPT-4o**											
	1	0.998 (0.994-1.000)		0.784 (0.765-0.803)		0.792 (0.774-0.811)		126 (121-132)		175 (165-187)	
	2	0.991 (0.985-0.996)		0.809 (0.794-0.824)		0.822 (0.808-0.838)		126 (121-132)		159 (150-167)	
	3	0.979 (0.970-0.986)		0.796 (0.778-0.813)		0.817 (0.802-0.833)		126 (121-132)		157 (149-166)	
**GPT-4o-mini**											
	1	0.945 (0.933-0.956)		0.835 (0.820-0.849)		0.878 (0.863-0.894)		126 (121-132)		141 (133-149)	
	2	0.918 (0.903-0.931)		0.822 (0.805-0.838)		0.885 (0.871-0.900)		126 (121-132)		134 (127-141)	
	3	0.886 (0.868-0.903)		0.791 (0.771-0.809)		0.880 (0.864-0.894)		126 (121-132)		130 (123-137)	
**Gemini 2.0 Flash**											
	1	0.999 (0.999-1.000)		0.631 (0.607-0.656)		0.639 (0.616-0.667)		126 (121-132)		262 (245-277)	
	2	0.996 (0.990-0.999)		0.719 (0.699-0.740)		0.728 (0.708-0.750)		126 (121-132)		202 (188-214)	
	3	0.993 (0.986-0.998)		0.722 (0.701-0.743)		0.731 (0.711-0.751)		126 (121-132)		197 (185-210)	
**Gemma 3**											
	1	0.995 (0.990-0.998)		0.531 (0.506-0.558)		0.541 (0.514-0.571)		126 (121-132)		332 (309-353)	
	2	0.993 (0.987-0.997)		0.621 (0.596-0.647)		0.631 (0.605-0.658)		126 (121-132)		270 (250-288)	
	3	0.989 (0.983-0.994)		0.634 (0.611-0.656)		0.647 (0.623-0.673)		126 (121-132)		254 (235-270)	
**Qwen2.5-VL-72B**											
	1	0.919 (0.903-0.934)		0.765 (0.741-0.784)		0.827 (0.806-0.851)		126 (121-132)		166 (155-178)	
	2	0.879 (0.857-0.899)		0.752 (0.729-0.774)		0.846 (0.826-0.867)		126 (121-132)		152 (140-162)	
	3	0.847 (0.823-0.869)		0.736 (0.714-0.757)		0.854 (0.837-0.875)		126 (121-132)		139 (130-148)	
**Qwen-2.5VL-7B**											
	1	0.929 (0.914-0.943)		0.425 (0.401-0.450)		0.462 (0.436-0.493)		126 (121-132)		382 (361-400)	
	2	0.881 (0.861-0.902)		0.470 (0.445-0.493)		0.531 (0.503-0.559)		126 (121-132)		319 (299-338)	
	3	0.860 (0.837-0.884)		0.475 (0.449-0.501)		0.547 (0.520-0.580)		126 (121-132)		299 (278-319)	

^a^IOU: intersection over union.

^b^PE: physical examination.

Of all models evaluated, GPT-4o, Gemini 2.0 Flash, and Gemma 3 achieved the highest recall (0.998, 0.999, and 0.995, respectively) at a sampling rate of 1. We selected GPT-4o as our optimal model due to its superior combination of high recall with the best IOU (0.784) and precision (0.792) and lowest predicted length (175 seconds) among high-recall performers.

At 1-second sampling, GPT-4o-mini showed significantly higher IOU than GPT-4o (mean 0.835, SD 0.180 vs mean 0.785, SD 0.220; mean difference 0.050, 95% CI 0.032-0.068; t_486_=5.49; Cohen *dz*=0.25; *P*<.001). Conversely, GPT-4o achieved significantly higher recall (mean 0.998, SD 0.036) than GPT-4o-mini (mean 0.945, SD 0.138; mean difference −0.052, 95% CI −0.064 to −0.041; t_488_=−8.66; Cohen *dz*=−0.39; *P*<.001), representing a precision-recall trade-off (Multimedia Appendix 3).

We observed that increasing sampling rates generally reduced recall while improving IOU and precision. This inverse relationship between sampling rate and recall demonstrates how sampling frequency affects detection performance, with more frequent sampling capturing more examination events but potentially introducing more false positives. Using bootstrap median HMM matrices did not materially change performance at 1-second sampling (Δrecall≤0.002; ΔIOU≤0.003; Multimedia Appendix 1).

### Classification Distribution Analysis

**Figure 4:**
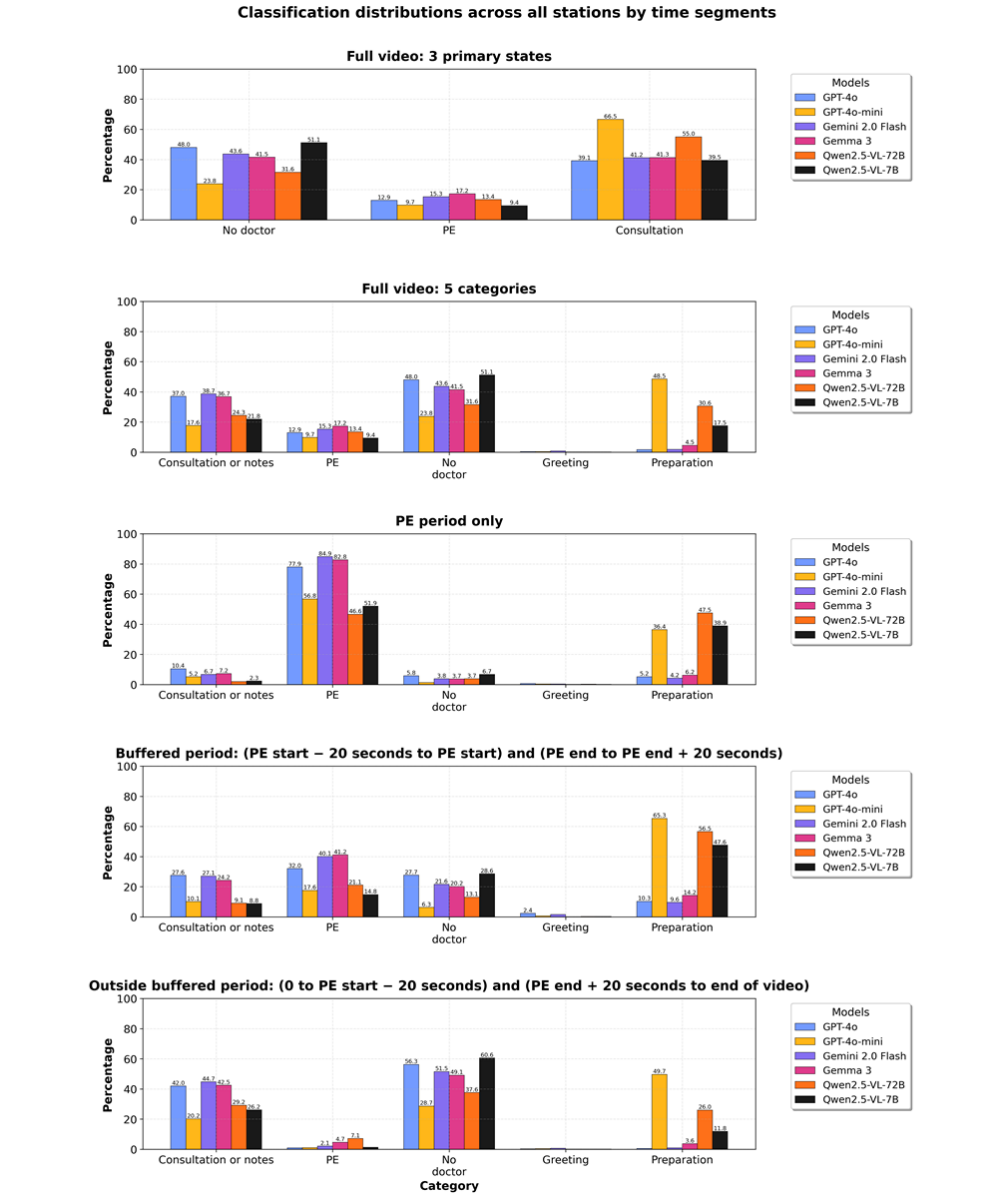
Classification distribution percentages by station: taking notes or consulting, physical examination (PE), no doctor, greeting, and preparation.

Figure 4 illustrates classification distributions across different time segments, revealing distinct patterns among the models. In the analysis of the 3 primary states, all models showed relatively consistent classification patterns, with GPT-4o-mini and Qwen2.5-VL-72B notably classifying more “no doctor” segments as “preparation” compared to other models.

During PE periods, all models correctly identified a high percentage of frames as “physical examination.” The buffer period analysis (–20 seconds to +20 seconds) showed a gradual transition between states, whereas classifications outside the buffered period correctly showed minimal “physical examination” identifications, confirming the models’ spatial accuracy.

### Station-Specific Performance at 1 Frame Per Second

**Figure 5:**
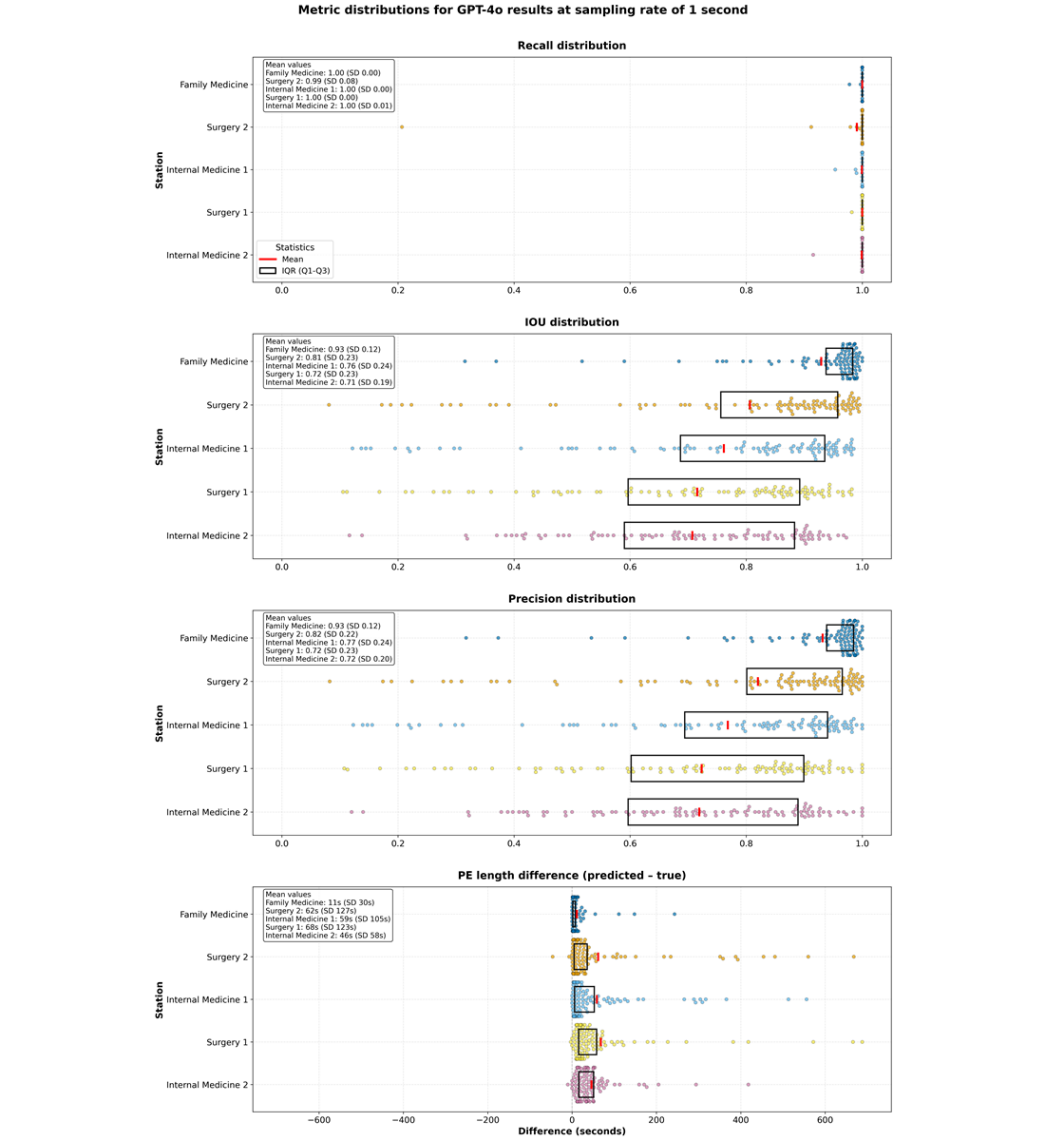
Recall, intersection over union (IOU), and physical examination (PE) length difference distributions with IQRs by station for GPT-4o.

We examined performance variations across the 5 different OSCE station types to evaluate our approach’s adaptability to different clinical scenarios. Figure 5 shows that GPT-4o achieved high recall performance across all stations, with most cases achieving near-perfect recall (100%).

The family medicine station exhibited the highest IOU (mean 0.93), likely because this station did not require medical students to place patients in a supine position for assessment, which reduced false positive PE classifications. The surgery 1 and internal medicine 2 stations showed the lowest IOU means (0.72 and 0.71, respectively), although they still maintained strong performance.

The PE length difference analysis in the bottom panel of Figure 5 indicates that GPT-4o generally predicted slightly longer examination periods than the ground truth across all stations, with mean differences ranging from 11 seconds (family medicine) to 68 seconds (surgery 1). These extended predictions ensure a comprehensive capture of examination activities while maintaining manageable review segments.

### Classification Error Analysis

We identified 2 primary types of error cases that affected our models’ performance: false negatives that reduced recall and false positives that reduced precision and IOU. Table 3 categorizes these errors into 5 distinct failure modes with representative examples.

**Table 3:** Classification failure mode analysis.

Case		Image	Model output and analysis
**Out-of-frame examination**			
	Error type: false negative Model classification: “no doctor” True classification: “physical examination”	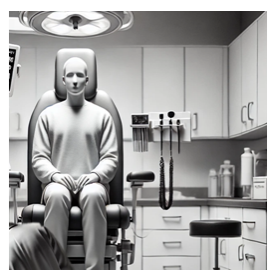	Model output: the patient is sitting on the chair. There is no doctor in the image. Failure reasoning: the camera fails to capture the full examination that takes place around the legs of the patient.
**Patient view occlusion**			
	Error type: false negative Model classification: “consulting” True classification: “physical examination”	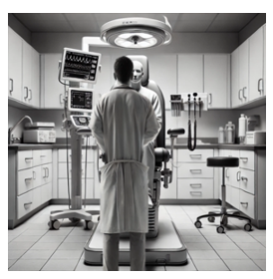	Model output: the patient is sitting on the chair. The doctor is consulting the patient. Failure reasoning: the medical student is obstructing the view of the patient, while the doctor is palpating the hands of the patient.
**Verbal instruction examinations**			
	Error type: false negative Model classification: “consulting” True classification: “physical examination”	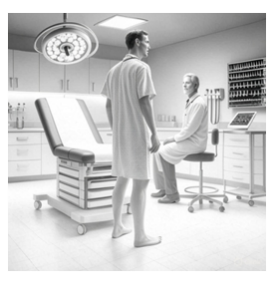	Model output: the doctor appears to be seated and observing the patient, possibly talking with the patient. Failure reasoning: without the audio, it is very difficult to differentiate a patient walking to the medical chair and a patient being tested on their gait.
**Preparation activity**			
	Error type: false positive Model classification: “physical examination” True classification: “preparation”	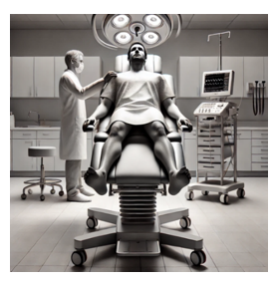	Model output: the doctor is standing up next to the patient, appearing to examine or interact with the patient’s shoulders, indicating a physical examination is taking place. Failure reasoning: while the doctor is lowering the chair for the patient, the model believes that the medical student is assessing the shoulders of the patient due to the student’s hand position on the patient.
**Bird’s eye view**			
	Error type: false positive Model classification: “physical examination” True classification: “consulting”	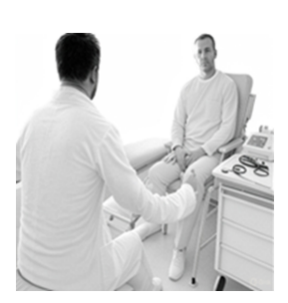	Model output: the doctor is crouched in front of the patient, appearing to examine or interact with the patient’s lower body, indicating a physical examination is taking place. Failure reasoning: due to the camera being positioned higher than the eye level, if the medical student is close to the patient while taking notes with their hands hovering over the patient’s legs while they explain something to the patient, the model thinks that the student is assessing the patient’s legs from their hand position on the patient’s legs.

All images used in the error analysis (Table 3) were generated by Grok 3 (xAI) to protect patient and student privacy while accurately representing the classification challenges observed in the actual OSCE videos.

These failure modes provide valuable insights for future system optimizations, particularly regarding camera placement and the potential integration of additional contextual information.

## Discussion

### Principal Findings

This study demonstrates that integrating MM-LLMs with temporal modeling provides an effective solution for automatically segmenting PE periods within OSCE videos. Our approach achieved 99.8% recall while reducing the video content requiring review by 81%, offering substantial benefits for both manual and automated assessment of medical student clinical skills. In the following sections, we discuss the implications, limitations, and future directions of this work.

The emission matrix (Figure 2B) showed 63.5% frame-level accuracy for “physical examination” states, with 31.8% misclassified as “conversation” due to similar visual features between these activities. However, high self-transition probabilities (0.857 for “physical examination”) enabled Viterbi decoding to enforce temporal consistency and produce accurate segmentations despite frame-level noise, demonstrating the necessity of temporal modeling for this task.

### Significance for Medical Education Assessment

The high recall rate (99.8%) achieved by our GPT-4o implementation represents a critical advancement for clinical skill assessment. In medical education, missing even brief examination components can lead to incomplete evaluations, potentially overlooking critical errors or skillful techniques that determine pass or fail outcomes. Our approach ensures comprehensive capture of examination activities while substantially reducing the review burden.

By reducing a 15-minute (900-second) video to an average of 175 seconds of relevant content, our method creates significant efficiency gains for educational institutions. For programs conducting OSCEs with hundreds of students across multiple stations, this could translate to significant time savings, potentially thousands of hours saved annually. Beyond time savings, this approach enhances assessment quality by allowing evaluators to focus their attention on the most pertinent segments of student performance.

The station-specific performance analysis revealed important insights about contextual factors affecting detection accuracy. Family medicine stations achieved the highest precision (93.2%) and IOU (93.4%), likely due to their seated examination format with minimal patient repositioning. In contrast, the surgery stations showed more variability in precision (72.3%), reflecting the challenges posed by frequent patient repositioning and varying examination techniques. These findings highlight how PE contexts influence detection performance and suggest potential optimization strategies for different clinical scenarios.

### Cost-Benefit Consideration

When comparing model configurations, GPT-4o achieved near-exhaustive capture of PE frames (recall=0.998) with lower IOU (0.784) at approximately US $1.25 per 15-minute video, whereas GPT-4o-mini yielded lower recall (0.945) but higher IOU (0.835) at approximately US $0.06 per video. Interpreted for deployment, GPT-4o minimizes the risk of missing examination content and is preferable when recall is paramount (eg, high-stakes evaluations), whereas GPT-4o-mini offers a near 20-fold cost reduction and yields more conservative segments that can streamline review. Selection of the operating point should reflect local tolerance for missed frames, desired review efficiency, and budget constraints.

### Practical Implementation

Implementation in educational settings should begin with optimizing camera positioning. Our error analysis identified several failure modes directly attributable to suboptimal camera angles, including out-of-frame examinations and patient view occlusion. On the basis of these findings, we recommend positioning cameras at eye level with a side view that captures both the medical student and patient simultaneously.

Institutions must also consider data privacy regulations when implementing artificial intelligence–based video analysis systems. Our research used HIPAA- and FERPA-compliant application programming interfaces for proprietary models, whereas open-source alternatives were hosted on secure institutional infrastructure. These approaches demonstrate viable pathways for maintaining compliance while leveraging advanced artificial intelligence capabilities in educational settings.

### Limitations

This study was conducted at a single institution with specific room layouts, camera positions, and station designs, which may limit generalizability to other educational settings and recording environments. Although this approach consistently identified PE segments, some dynamic or verbally cued maneuvers (eg, gait examination) remain challenging when relying on video frames alone; incorporating audio or transcripts may help resolve these issues. The current system detects the presence of a PE rather than classifying specific techniques, which constrains immediate use for fine-grained skill feedback. While annotations underwent quality checks, we did not conduct a formal interrater reliability study, so boundary uncertainty in the reference labels may persist.

With respect to human diversity and fairness, all recordings originated from a single site. Model behavior may vary across populations that differ in skin tone, body habitus, attire, assistive devices, or interaction styles, and we did not stratify performance by these attributes. Multisite validation with prespecified subgroup analyses will be necessary to quantify any performance differences and guide mitigation strategies before broad deployment.

### Recommendations and Future Work

Future work should extend this PE and non-PE segmentation to finer-grained, technique-level labels that align with OSCE rubrics (eg, inspection, palpation, percussion, and auscultation). Moving beyond binary segmentation would make outputs immediately actionable for feedback and remediation. Methodologically, integrating audio or transcripts with video is a logical next step, particularly for dynamic or verbally cued maneuvers; multimodal fusion may reduce boundary errors and improve recognition of short, transient actions. In parallel, interface and workflow studies are needed to evaluate how predicted segments affect reviewer behavior, interrater reliability, and total review time in authentic grading settings.

External validation across multiple sites should quantify generalizability and fairness by reporting subgroup performance and CIs on prespecified attributes such as skin tone, body habitus, attire, and assistive devices. Where performance differences are observed, prompt design, postprocessing, or sampling strategies could be adapted to mitigate disparities. Prospective studies should also formalize decision criteria for selecting the model family and sampling rate using an explicit utility framework that balances recall (risk of missed PE content), precision and IOU (review efficiency), and monetary cost; these studies can incorporate paired statistical tests to determine whether observed differences between model configurations are significant in the target deployment context. Finally, releasing the code for the temporal postprocessing pipeline and standardized prompts, together with clear governance and consent procedures, would support reproducibility and accelerate comparative evaluations.

### Conclusions

In a large corpus of OSCE recordings, a zero-shot MM-LLM paired with lightweight temporal decoding reliably isolated PE periods while substantially reducing the duration of video requiring human review. This approach meets the study aim of testing whether PE segments can be identified without previous task-specific training to lessen evaluation burden. Although this work is limited to a single site and targets PE presence rather than specific techniques, sensitivity analyses support the robustness of the temporal model, and the results collectively indicate that selective review guided by model-predicted segments is a practical pathway for scaling assessment in medical education. Broader, multisite validation with attention to fairness, together with extensions to technique-level labeling and multimodal inputs, are appropriate next steps before routine implementation.

## Appendix

Multimedia Appendix 1 Performance metrics of artificial intelligence models across sampling rates and buffer times with 95% CIs.

Multimedia Appendix 2 Hidden Markov model parameter bootstrap stability.

Multimedia Appendix 3 Paired statistical comparisons of GPT-4o-mini versus GPT-4o performance metrics.

## Abbreviations

FERPA: Family Educational Rights and Privacy Act
HIPAA: Health Insurance Portability and Accountability Act
HMM: hidden Markov model
IOU: intersection over union
MM-LLM: multimodal large language model
OSCE: objective structured clinical examination
PE: physical examination
UT: University of Texas

## Data Availability

The datasets generated or analyzed during this study are not publicly available because they comprise Family Educational Rights and Privacy Act–protected objective structured clinical examination recordings with identifiable audio and video but are available from the corresponding author on reasonable request.
