# Supplementary material for: Physical Examination Identification in Medical Education Videos: Zero-Shot Multimodal AI With Temporal Sequence Optimization Study
Source: JMIR AI. 2025 Dec 18;4:e76586. doi: 10.2196/76586 (PMC12757708; doi:10.2196/76586)
Supplement: Multimedia Appendix 1 [file ai_v4i1e76586_app1.docx]

| **Appendix 1. HMM Parameter Bootstrap Stability** | | | | | | |
| --- | --- | --- | --- | --- | --- | --- |
| **Matrix Type** | **Matrix Element** | **Bootstrap Mean** | **Bootstrap SD** | **95% CI Lower** | **95% CI Upper** | **CI Width** |
| **Transition** | **No Doctor → No Doctor** | **0.970** | **0.011** | **0.945** | **0.989** | **0.044** |
| **Transition** | **Consultation → Consultation** | **0.924** | **0.013** | **0.901** | **0.952** | **0.050** |
| **Transition** | **PE → PE** | **0.857** | **0.011** | **0.833** | **0.876** | **0.043** |
| **Emission** | **No Doctor → No Doctor** | **0.940** | **0.012** | **0.918** | **0.963** | **0.045** |
| **Emission** | **Consultation → Consultation** | **0.852** | **0.014** | **0.826** | **0.878** | **0.052** |
| **Emission** | **PE → PE** | **0.635** | **0.025** | **0.589** | **0.683** | **0.094** |

Mean Absolute Change (MAC) = 0.008 for both transition and emission matrices, indicating excellent stability.
