# Supplementary material for: Physical Examination Identification in Medical Education Videos: Zero-Shot Multimodal AI With Temporal Sequence Optimization Study
Source: JMIR AI. 2025 Dec 18;4:e76586. doi: 10.2196/76586 (PMC12757708; doi:10.2196/76586)
Supplement: Multimedia Appendix 2 [file ai_v4i1e76586_app2.docx]

| **Appendix 1. Performance Metrics with Buffer with 95% Confidence Intervals** | | | | | | | |
| --- | --- | --- | --- | --- | --- | --- | --- |
| **Model** | **Sampling Rate** | **Buffer** | **Recall** | **IOU** | **Precision** | **PE Length (sec)** | **Predicted Length (sec)** |
| **GPT-4o** | **1** | **0s** | **0.998 [0.994, 1.000]** | **0.784 [0.765, 0.803]** | **0.792 [0.774, 0.811]** | **126 [121, 132]** | **175 [165, 187]** |
| **GPT-4o** | **1** | **15s** | **0.999 [0.998, 1.000]** | **0.654 [0.637, 0.670]** | **0.664 [0.648, 0.680]** | **126 [121, 132]** | **202 [192, 214]** |
| **GPT-4o** | **1** | **30s** | **1.000 [1.000, 1.000]** | **0.559 [0.545, 0.573]** | **0.571 [0.556, 0.586]** | **126 [121, 132]** | **232 [221, 243]** |
| **GPT-4o** | **2** | **0s** | **0.991 [0.985, 0.996]** | **0.809 [0.794, 0.824]** | **0.822 [0.808, 0.838]** | **126 [121, 132]** | **159 [150, 167]** |
| **GPT-4o** | **2** | **15s** | **0.996 [0.993, 0.999]** | **0.684 [0.670, 0.697]** | **0.696 [0.682, 0.711]** | **126 [121, 132]** | **184 [176, 193]** |
| **GPT-4o** | **2** | **30s** | **0.999 [0.997, 1.000]** | **0.584 [0.571, 0.598]** | **0.597 [0.584, 0.610]** | **126 [121, 132]** | **213 [205, 222]** |
| **GPT-4o** | **3** | **0s** | **0.979 [0.970, 0.986]** | **0.796 [0.778, 0.813]** | **0.817 [0.802, 0.833]** | **126 [121, 132]** | **157 [149, 166]** |
| **GPT-4o** | **3** | **15s** | **0.987 [0.980, 0.993]** | **0.688 [0.672, 0.703]** | **0.703 [0.689, 0.719]** | **126 [121, 132]** | **180 [172, 189]** |
| **GPT-4o** | **3** | **30s** | **0.993 [0.987, 0.997]** | **0.591 [0.576, 0.605]** | **0.604 [0.590, 0.619]** | **126 [121, 132]** | **210 [201, 219]** |
| **GPT-4o-mini** | **1** | **0s** | **0.945 [0.933, 0.956]** | **0.835 [0.820, 0.849]** | **0.878 [0.863, 0.894]** | **126 [121, 132]** | **141 [133, 149]** |
| **GPT-4o-mini** | **1** | **15s** | **0.974 [0.963, 0.982]** | **0.719 [0.704, 0.733]** | **0.737 [0.721, 0.753]** | **126 [121, 132]** | **168 [161, 176]** |
| **GPT-4o-mini** | **1** | **30s** | **0.984 [0.975, 0.991]** | **0.616 [0.603, 0.630]** | **0.628 [0.613, 0.643]** | **126 [121, 132]** | **198 [190, 205]** |
| **GPT-4o-mini** | **2** | **0s** | **0.918 [0.903, 0.931]** | **0.822 [0.805, 0.838]** | **0.885 [0.871, 0.900]** | **126 [121, 132]** | **134 [127, 141]** |
| **GPT-4o-mini** | **2** | **15s** | **0.955 [0.943, 0.965]** | **0.728 [0.714, 0.742]** | **0.757 [0.741, 0.772]** | **126 [121, 132]** | **159 [152, 167]** |
| **GPT-4o-mini** | **2** | **30s** | **0.974 [0.963, 0.982]** | **0.632 [0.618, 0.644]** | **0.647 [0.631, 0.662]** | **126 [121, 132]** | **188 [181, 196]** |
| **GPT-4o-mini** | **3** | **0s** | **0.886 [0.868, 0.903]** | **0.791 [0.771, 0.809]** | **0.880 [0.865, 0.894]** | **126 [121, 132]** | **130 [123, 137]** |
| **GPT-4o-mini** | **3** | **15s** | **0.931 [0.916, 0.945]** | **0.723 [0.707, 0.737]** | **0.767 [0.752, 0.782]** | **126 [121, 132]** | **153 [146, 161]** |
| **GPT-4o-mini** | **3** | **30s** | **0.959 [0.947, 0.970]** | **0.636 [0.621, 0.650]** | **0.658 [0.643, 0.673]** | **126 [121, 132]** | **183 [175, 190]** |
| **Gemini-2.0-Flash** | **1** | **0s** | **0.999 [0.999, 1.000]** | **0.631 [0.607, 0.656]** | **0.639 [0.612, 0.667]** | **126 [121, 132]** | **262 [245, 277]** |
| **Gemini-2.0-Flash** | **1** | **15s** | **1.000 [1.000, 1.000]** | **0.536 [0.516, 0.557]** | **0.546 [0.527, 0.569]** | **126 [121, 132]** | **289 [273, 305]** |
| **Gemini-2.0-Flash** | **1** | **30s** | **1.000 [1.000, 1.000]** | **0.465 [0.449, 0.483]** | **0.477 [0.460, 0.496]** | **126 [121, 132]** | **318 [302, 334]** |
| **Gemini-2.0-Flash** | **2** | **0s** | **0.996 [0.990, 0.999]** | **0.719 [0.699, 0.740]** | **0.728 [0.706, 0.750]** | **126 [121, 132]** | **202 [188, 214]** |
| **Gemini-2.0-Flash** | **2** | **15s** | **0.998 [0.993, 1.000]** | **0.613 [0.597, 0.632]** | **0.624 [0.606, 0.640]** | **126 [121, 132]** | **227 [214, 240]** |
| **Gemini-2.0-Flash** | **2** | **30s** | **0.998 [0.995, 1.000]** | **0.527 [0.513, 0.543]** | **0.540 [0.524, 0.557]** | **126 [121, 132]** | **257 [243, 269]** |
| **Gemini-2.0-Flash** | **3** | **0s** | **0.993 [0.986, 0.998]** | **0.722 [0.701, 0.743]** | **0.731 [0.711, 0.751]** | **126 [121, 132]** | **197 [185, 210]** |
| **Gemini-2.0-Flash** | **3** | **15s** | **0.995 [0.989, 0.999]** | **0.623 [0.605, 0.642]** | **0.633 [0.616, 0.653]** | **126 [121, 132]** | **223 [209, 233]** |
| **Gemini-2.0-Flash** | **3** | **30s** | **0.996 [0.990, 1.000]** | **0.535 [0.520, 0.551]** | **0.547 [0.531, 0.563]** | **126 [121, 132]** | **250 [237, 263]** |
| **Gemma-3** | **1** | **0s** | **0.995 [0.990, 0.998]** | **0.531 [0.506, 0.558]** | **0.541 [0.514, 0.571]** | **126 [121, 132]** | **332 [309, 353]** |
| **Gemma-3** | **1** | **15s** | **0.997 [0.993, 1.000]** | **0.460 [0.439, 0.482]** | **0.470 [0.449, 0.495]** | **126 [121, 132]** | **359 [336, 380]** |
| **Gemma-3** | **1** | **30s** | **0.998 [0.995, 1.000]** | **0.405 [0.387, 0.423]** | **0.416 [0.399, 0.437]** | **126 [121, 132]** | **387 [365, 409]** |
| **Gemma-3** | **2** | **0s** | **0.993 [0.987, 0.997]** | **0.621 [0.596, 0.647]** | **0.631 [0.605, 0.658]** | **126 [121, 132]** | **270 [250, 288]** |
| **Gemma-3** | **2** | **15s** | **0.996 [0.991, 0.999]** | **0.538 [0.516, 0.559]** | **0.548 [0.526, 0.569]** | **126 [121, 132]** | **295 [275, 314]** |
| **Gemma-3** | **2** | **30s** | **0.997 [0.992, 1.000]** | **0.468 [0.449, 0.487]** | **0.479 [0.460, 0.497]** | **126 [121, 132]** | **324 [304, 342]** |
| **Gemma-3** | **3** | **0s** | **0.989 [0.983, 0.994]** | **0.634 [0.611, 0.656]** | **0.647 [0.623, 0.673]** | **126 [121, 132]** | **254 [235, 270]** |
| **Gemma-3** | **3** | **15s** | **0.994 [0.989, 0.998]** | **0.554 [0.534, 0.574]** | **0.567 [0.546, 0.588]** | **126 [121, 132]** | **277 [258, 293]** |
| **Gemma-3** | **3** | **30s** | **0.997 [0.993, 0.999]** | **0.481 [0.464, 0.499]** | **0.494 [0.476, 0.512]** | **126 [121, 132]** | **306 [287, 322]** |
| **Qwen-2.5VL-72b** | **1** | **0s** | **0.919 [0.903, 0.934]** | **0.765 [0.741, 0.785]** | **0.827 [0.806, 0.851]** | **126 [121, 132]** | **166 [155, 178]** |
| **Qwen-2.5VL-72b** | **1** | **15s** | **0.956 [0.943, 0.967]** | **0.673 [0.654, 0.691]** | **0.701 [0.682, 0.722]** | **126 [121, 132]** | **193 [182, 205]** |
| **Qwen-2.5VL-72b** | **1** | **30s** | **0.974 [0.963, 0.983]** | **0.586 [0.568, 0.603]** | **0.604 [0.585, 0.622]** | **126 [121, 132]** | **222 [211, 234]** |
| **Qwen-2.5VL-72b** | **2** | **0s** | **0.879 [0.857, 0.899]** | **0.752 [0.729, 0.774]** | **0.846 [0.828, 0.867]** | **126 [121, 132]** | **152 [140, 162]** |
| **Qwen-2.5VL-72b** | **2** | **15s** | **0.925 [0.906, 0.940]** | **0.681 [0.663, 0.698]** | **0.729 [0.712, 0.748]** | **126 [121, 132]** | **177 [165, 187]** |
| **Qwen-2.5VL-72b** | **2** | **30s** | **0.953 [0.939, 0.965]** | **0.602 [0.586, 0.617]** | **0.629 [0.612, 0.647]** | **126 [121, 132]** | **206 [194, 216]** |
| **Qwen-2.5VL-72b** | **3** | **0s** | **0.847 [0.823, 0.869]** | **0.736 [0.714, 0.757]** | **0.854 [0.837, 0.875]** | **126 [121, 132]** | **139 [130, 148]** |
| **Qwen-2.5VL-72b** | **3** | **15s** | **0.895 [0.874, 0.912]** | **0.679 [0.661, 0.697]** | **0.747 [0.729, 0.765]** | **126 [121, 132]** | **162 [153, 171]** |
| **Qwen-2.5VL-72b** | **3** | **30s** | **0.934 [0.917, 0.947]** | **0.610 [0.595, 0.626]** | **0.647 [0.629, 0.665]** | **126 [121, 132]** | **191 [182, 200]** |
| **Qwen-2.5VL-7b** | **1** | **0s** | **0.929 [0.914, 0.943]** | **0.425 [0.401, 0.450]** | **0.462 [0.436, 0.493]** | **126 [121, 132]** | **382 [364, 400]** |
| **Qwen-2.5VL-7b** | **1** | **15s** | **0.958 [0.946, 0.969]** | **0.394 [0.374, 0.416]** | **0.416 [0.393, 0.442]** | **126 [121, 132]** | **407 [387, 426]** |
| **Qwen-2.5VL-7b** | **1** | **30s** | **0.973 [0.963, 0.982]** | **0.361 [0.343, 0.381]** | **0.376 [0.356, 0.398]** | **126 [121, 132]** | **434 [414, 452]** |
| **Qwen-2.5VL-7b** | **2** | **0s** | **0.881 [0.861, 0.902]** | **0.470 [0.445, 0.493]** | **0.531 [0.503, 0.559]** | **126 [121, 132]** | **319 [299, 338]** |
| **Qwen-2.5VL-7b** | **2** | **15s** | **0.918 [0.900, 0.935]** | **0.444 [0.421, 0.465]** | **0.479 [0.454, 0.503]** | **126 [121, 132]** | **343 [323, 362]** |
| **Qwen-2.5VL-7b** | **2** | **30s** | **0.946 [0.929, 0.960]** | **0.410 [0.389, 0.429]** | **0.431 [0.409, 0.453]** | **126 [121, 132]** | **370 [351, 389]** |
| **Qwen-2.5VL-7b** | **3** | **0s** | **0.860 [0.837, 0.884]** | **0.475 [0.449, 0.504]** | **0.547 [0.519, 0.577]** | **126 [121, 132]** | **299 [278, 320]** |
| **Qwen-2.5VL-7b** | **3** | **15s** | **0.897 [0.876, 0.918]** | **0.452 [0.429, 0.477]** | **0.499 [0.472, 0.527]** | **126 [121, 132]** | **321 [300, 341]** |
| **Qwen-2.5VL-7b** | **3** | **30s** | **0.924 [0.905, 0.942]** | **0.419 [0.399, 0.441]** | **0.449 [0.425, 0.474]** | **126 [121, 132]** | **348 [327, 367]** |
