# Supplementary material for: Physical Examination Identification in Medical Education Videos: Zero-Shot Multimodal AI With Temporal Sequence Optimization Study
Source: JMIR AI. 2025 Dec 18;4:e76586. doi: 10.2196/76586 (PMC12757708; doi:10.2196/76586)
Supplement: Multimedia Appendix 3 [file ai_v4i1e76586_app3.docx]

| **Appendix 3. Paired Statistical Comparisons of GPT-4o-mini vs GPT-4o Performance Metrics** | | | | | | | | |  |
| --- | --- | --- | --- | --- | --- | --- | --- | --- | --- |
| **Sampling Rate** | **Metric** | **Count** |  | **Mean (SD)** | | **95% CI** | **p-value** | **dz** |  |
|  |  |  |  | **GPT-4o-mini** | **GPT-4o** |  |  |  |  |
| **1s** | **IOU** | **500** |  | **0.835 (0.180)** | **0.785 (0.220)** | **[0.032, 0.068]** | **<0.0001** | **0.25** |  |
| **1s** | **Recall** | **500** |  | **0.945 (0.138)** | **0.998 (0.036)** | **[-0.064, -0.041]** | **<0.0001** | **-0.39** |  |
| **2s** | **IOU** | **100** |  | **0.822 (0.178)** | **0.812 (0.179)** | **[-0.006, 0.026]** | **0.215** | **0.06** |  |
| **2s** | **Recall** | **100** |  | **0.918 (0.173)** | **0.991 (0.058)** | **[-0.088, -0.059]** | **<0.0001** | **-0.45** |  |
| **3s** | **IOU** | **100** |  | **0.791 (0.200)** | **0.798 (0.185)** | **[-0.026, 0.011]** | **0.123** | **-0.04** |  |
| **3s** | **Recall** | **100** |  | **0.886 (0.208)** | **0.979 (0.100)** | **[-0.109, 0.076]** | **<0.0001** | **-0.49** |  |

*dz = Cohen's dz effect size
